# Leukocytes Ratios in Feline Systemic Inflammatory Response Syndrome and Sepsis: A Retrospective Analysis of 209 Cases

**DOI:** 10.3390/ani11061644

**Published:** 2021-06-01

**Authors:** Eleonora Gori, Alessio Pierini, Ilaria Lippi, George Lubas, Veronica Marchetti

**Affiliations:** Department of Veterinary Sciences, Veterinary Teaching Hospital “Mario Modenato”, University of Pisa, 56122 Pisa, Italy; Eleonora.gori@vet.unipi.it (E.G.); ilaria.lippi@unipi.it (I.L.); george.lubas@unipi.it (G.L.); veronica.marchetti@unipi.it (V.M.)

**Keywords:** cat, inflammation, white blood cells, NLR, neutrophil-to-lymphocyte ratio

## Abstract

**Simple Summary:**

Feline sepsis is a life-threatening syndrome in which blood diagnostic and prognostic markers are limited. We investigated the differences in neutrophil-to-lymphocyte ratio and other white-blood-cell ratios between healthy and sick cats (systemic inflammation (SIRS) and/or septic cats) and then the use of these ratios as prognostic markers. This study included 76 healthy cats (blood-donors), 54 cats with SIRS and 79 cats with sepsis. A cat with an NLR > 4.53 had a 44-fold chance to have SIRS or sepsis, although only BLR and BNLR were different between SIRS and sepsis groups. The NLR has been shown as a prognostic marker in sick cats. This is a novel investigation about leukocyte ratios in the cat, and the NLR may be used as a prognostic parameter in cats with SIRS or sepsis, and BLR and BNLR demonstrate themselves as promising tools in differentiating SIRS from sepsis.

**Abstract:**

Sepsis is a challenging condition in which hematological prognostic and diagnostic markers in cats are limited. The aims of this study were to test if there are any differences in leukocyte ratios (NLR, BLR and BNLR) between healthy, SIRS and septic cats (sick cats), and if, within sick cats, NLR, BLR and BNLR may be prognostic markers. A retrospective medical database study included 76 healthy cats (blood-donors), 54 SIRS and 79 septic cats. SIRS group was defined if cats fulfilled SIRS criteria. Sepsis was confirmed with an infectious focus on cytology or a positive culture for bacterial infection. Leukocyte ratios were compared among the three study groups and between survivors and non-survivors in sick cats. NLR resulted significantly higher in the sick group compared to healthy cats (*p* < 0.0001), although NLR was not different between SIRS and sepsis. An NLR > 4.53 had a sensitivity of 76% and a specificity of 93.4% to detect SIRS/sepsis (OR 44.8 95%CI 17–107). Only BLR and BNLR were significantly different between SIRS and sepsis. NLR was associated with mortality in the sick group (*p* = 0.04). Although NLR resulted higher in sick cats than healthy, BLR and BNLR demonstrated as promising tools in differentiating SIRS from sepsis. NLR was associated with mortality in sick cats.

## 1. Introduction

Despite sepsis is a challenging and well-studied condition in humans, hematological prognostic and diagnostic markers in cats are limited or based on small population studies. Most sepsis hematological markers, especially leukocytes ratios, are conducted on dogs [1,2,3,4,5]. In cats, in the last 20 years of literature, hematological markers have been studied in a few studies [6,7,8,9,10,11]. The first two papers [6,7] described the hematological features of a population of feline septic patients. They both highlighted that leukocyte count and band neutrophils might be altered in this type of patients, even if a percentage of them may display no WBC abnormalities [7,11], even compared to healthy controls and cats with systemic inflammatory response syndrome (SIRS) [7]. More recently, some authors studied serum amyloid A (SAA) in healthy cats compared to SIRS and septic cats [8]. This study confirmed that SAA might be a reliable marker to establish the presence of an inflammatory process, being higher also in the septic group compared to the SIRS one, although with a moderate ability to identify septic cats [8]. However, SAA was not able to predict cats’ mortality [8].

In 2020, two other studies considered new feline sepsis markers, as procalcitonin and heparin-binding protein levels [11] and cytokine and chemokine profile [10]. In the first study, the median procalcitonin concentration resulted significantly higher in the sepsis group than in the control group, although heparin-binding protein levels were not different. The other study on cytokine and chemokine showed that, even if some cytokines were undetectable in healthy and septic cats, other types of chemokines and cytokine may be helpful to differentiate sepsis from septic shock [10].

In humans, the neutrophil-to-lymphocyte ratio (NLR), as a readily accessible parameter that can be calculated using a complete blood count, has been found as an independent predictor of morbidity and mortality in sepsis, [12,13,14,15,16,17] and it has been also recently investigated in dogs [3,4,5]. The first study failed to find a significant difference in NLR between dogs with or without septic peritonitis and between survivors and non-survivors [3]. Afterwards, the NLR and other two leukocyte ratios (band neutrophil-to-neutrophil-to-lymphocyte ratio (BNLR) and band neutrophil-to-lymphocyte ratio (BLR)) were investigated in dogs with SIRS and sepsis [4,5]. In both reports, the NLR resulted lower in septic dogs than in SIRS dogs, although no significant associations between BNLR or BLR and the presence of sepsis or mortality were found [4,5].

We hypothesized that NLR, BLR and BNLR might be useful also in feline sepsis. As shown for dogs, leukocyte ratios may be suitable in differentiating healthy from SIRS and septic cats and, also, may be used as prognostic markers. The aims of this study were (1) to test if there are any differences in leukocytes ratios (NLR, BLR and BNLR) between healthy, SIRS and septic cats and (2) if, within sick cats, NLR, BLR and BNLR may be used as prognostic marker linked to the mortality.

## 2. Materials and Methods

The study population was composed of three cat populations: septic cats, systemic inflammatory response syndrome (SIRS) cats, and healthy cats. All these groups were searched using a retrospective medical records review from the database of the University of Pisa Veterinary Teaching Hospital.

Firstly, the electronic database was searched for cats with potential sepsis, using the terms “sepsis,” “septic inflammation,” and/or “positive bacterial culture” and “septic” in both clinical and cytology/culture records from January 2014 to January 2021. Records containing at least one of these words were reviewed to confirm the presence of documented sepsis. 

Secondly, medical records were then searched for cats with SIRS using “systemic inflammation,” “SIRS,” abnormal temperature, brady- and tachycardia and tachypnea. The SIRS group was defined if cats had at least three of the four following parameters: (1) abnormal temperature (≤37.8 or ≥39.7 °C); (2) tachycardia (≥225 beats/min) or bradycardia (≤140 beats/min); (3) tachypnea (≥40 breaths/min); and (4) WBC abnormalities (WBC ≥19500 or ≤5000 k/μL or band neutrophils ≥5%) [7]. Cats with SIRS and presenting an infectious focus, with intracytoplasmic bacteria, or positive culture, were recorded as septic [4,5,18]. Cats were excluded if sepsis was not confirmed from the medical record review based on the aforementioned criteria.

The final diagnosis of sepsis and SIRS group was recorded. Cats with uncountable leukocyte ratios because lymphocyte count was 0 were excluded from the study.

Lastly, the healthy group was composed only of blood-donor cats. Each cat included in the control group was affiliated with the blood donor program of the University. Blood donor cats were selected using the criteria indicated by the Ministry of Health Italian Guidelines: age range from 2 to 8 years, weight 5–7 kg, regularly vaccinated and did not receive any previous blood transfusion. At the time of donation, each animal was classified as healthy based on history, physical examination, hematobiochemical profile, urinalysis (including protein to creatinine ratio) and coprological exam. In addition, each cat had to test ELISA negative for both feline immunodeficiency virus (FIV) and feline leukemia virus (FeLV) [19].

CBC was performed in all cats at hospital admission using a laser cell counter (Procyte DX, IDEXX Laboratories, Westbrook, Maine, USA). The blood smear stained with May-Grunwald Giemsa (Aerospray Wescor, Delcon, Milan, Italy) was microscopically examined by experienced and trained clinical pathologists.

The leukocyte ratios were calculated as it follows:

NLR = (Segmented neutrophils + band neutrophils) ÷ lymphocytes,(1)

BLR = Band neutrophils ÷ lymphocytes,(2)

BNLR = (Band neutrophils ÷segmented neutrophils) ÷ lymphocytes,(3)

The presence of neutrophilic toxicity was recorded if cytoplasmic basophilia, cytoplasmic vacuolation, presence of Döhle bodies or toxic granulation were detected [20].

Sick cats (SIRS + sepsis groups) were divided into survivors and non-survivors, according to the discharge outcome, which was extrapolated from medical records: survivors (discharged from the hospital) and non-survivors (died during hospitalization).

Continuous variables were non-normally distributed and were presented as the median and interquartile range (IQR). Neutrophilic toxicity was evaluated as a categorical variable (present/absent). For the first aim, leukocyte ratios (NLR, BLR and BNLR) were compared among the three groups, using the Kruskal–Wallis test with pairwise comparisons with the Bonferroni correction test. If a statistically significant difference between SIRS and sepsis groups was found, a receiver operating characteristic curve (ROC) with the area under the curve (AUC) was calculated and the optimal cut-off to differentiate groups was assessed. The diagnostic cut-off and its sensitivity and specificity were determined according to the maximum Youden index. The presence of neutrophilic toxicity was compared between SIRS and septic cats using the Chi-square test. For the second aim, NLR, BLR and BNLR and the presence of neutrophilic toxicity were compared between survivors and non-survivors using a multivariable binary logistic regression model. In the model, independent parameters (neutrophilic toxicity, NLR, BLR and BNLR) were set as predictors and mortality as an outcome. Each variable with a *p*-value < 0.05 was considered independently associated with mortality. Statistical analysis was performed using IBM SPSS Statistics v.25 (IBM Corporation, New York, NY, USA), and a *p*-value <0.05 was considered statistically significant for the other tests.

## 3. Results

A total of 209 cats were retrospectively included. 

The septic group was composed of 79 cats. Most of the sepsis group was composed of domestic shorthairs (61/79; 77%). The remaining 18 cats belonged to the following breeds: Persian cat (five), Birman cat and Maine Coon (three cats each breed), Siamese and British shorthair (two cats each breed), Chartreux, Exotic shorthair and Ragdoll (one cat each breed). There were 30 females, 24 of which spayed, and 49 were males, 32 of them castrated. The underlying causes of sepsis were respiratory (*n* = 29), gastrointestinal (*n* = 21), nephro-urological (*n* = 13), cutaneous (*n* = 10), reproductive (*n* = 4) and post-trauma sepsis (*n* = 2). The median age was 5.4 years (IQR 7.2 years).

The SIRS group was composed of 54 cats. Forty-seven of the 54 cats (87%) were domestic shorthairs. The remaining seven cats were: Siberian, Chartreux and Persian cat (two cats each breed) and one Ragdoll. The SIRS group was composed of 17 females (13 of which spayed) and 35 males (23 castrated). The underlying SIRS causes were post-trauma (*n* = 20), gastrointestinal (*n* = 12), nephro-urological (*n* = 11), neoplastic (*n* = 3), respiratory, intoxication and infectious (2 cats each cause), neurological and cardiovascular (1 cat each cause). The median age was 6.2 years (IQR 8.8 years). 

The healthy group was constituted of 76 cats. The majority of the healthy group was composed of 54 domestic shorthairs, followed by 15 Maine Coons, 2 Siberians, 2 Bengal cats, 1 Chartreux, 1 Ragdoll and 1 Persian cat. The median age was 3.9 years (IQR 3.2 years). 

SIRS cats had a significantly higher median age (6.2 years) compared to sepsis and healthy groups (5.4 and 3.9 years, respectively) (*p* = 0.02).

**Figure 1:**
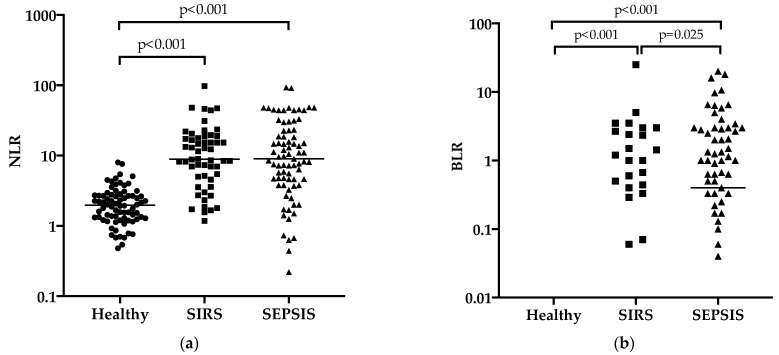

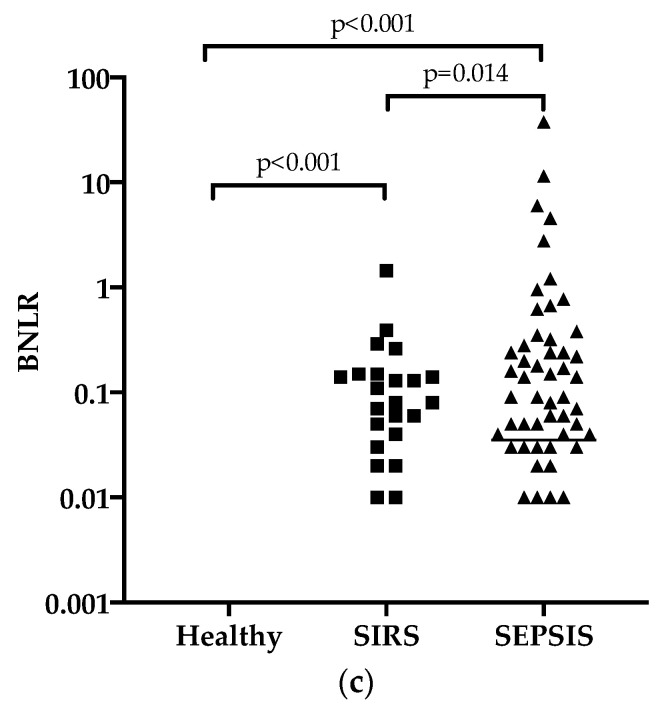
Scatterplots of neutrophil-to-lymphocyte ratio (NLR; **a**), band neutrophil-to-lymphocyte ratio (BLR; **b**) and band neutrophil-to-neutrophil-to-lymphocyte ratio (BNLR; **c**) in the three study groups. The leukocyte ratios are represented with a log10 scale on the Y-axis. In Figure 1a, the horizontal line represents the median value of NLR in the three groups. In Figure 1b–c, only the sepsis group had this horizontal line displayed as the median value since the median of the other groups is 0.

The values of leukocyte ratios are reported in Table 1. Briefly, NLR, BLR and BNLR resulted significantly different in SIRS and septic cats compared to healthy cats. BLR and BNLR were also significantly different between the SIRS and septic groups. Scatterplots of NLR, BLR and BNLR are presented in Figure 1.

**Figure 2:**
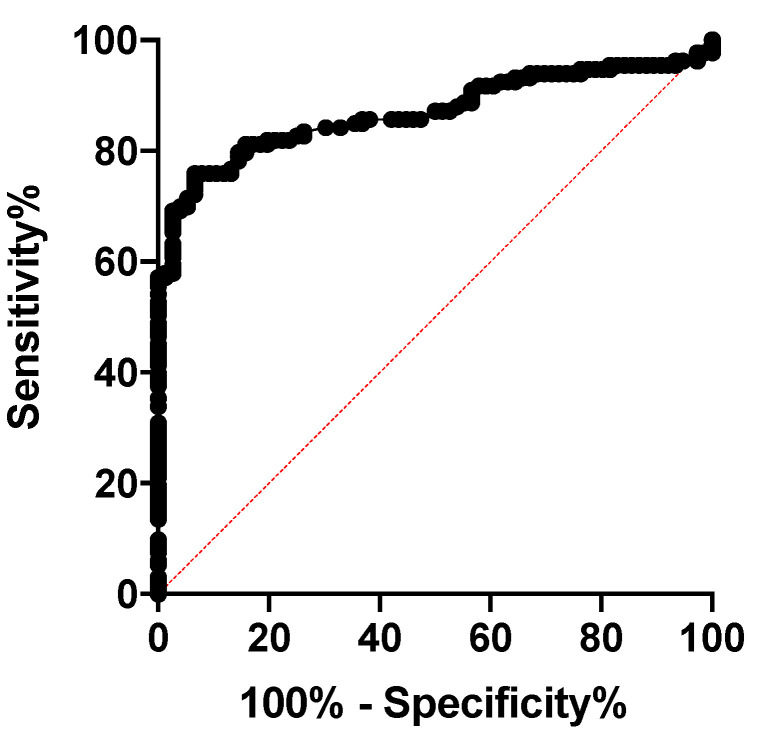
The receiver operating characteristic curve (ROC) analysis of the Neutrophil-to-lymphocyte ratio in healthy and sick cats (SIRS + sepsis cats) (*p* < 0.0001; AUC = 0.86 OR 44.8 95%CI 17–107).

Based on the ROC of NLR between healthy and sick cats (SIRS + sepsis cats), the best cut-off of NLR for SIRS and sepsis was 4.53 (*p* < 0.0001; OR 44.8 95%CI 17–107; Figure 2). In cats, an NLR greater than this threshold had a sensitivity of 76% and a specificity of 93.4% to detect SIRS and/or sepsis.

The ROC of BLR and BNLR resulted both statistically significant between SIRS and sepsis (*p* = 0.02; AUC 0.6 OR 2.45 95%CI 1.2–4.8 and *p* = 0.01; AUC = 0.63 OR 1.8 95%CI 0.9–3.8, respectively). Based on these results, the optimal cut-off points of BLR and BNLR for sepsis were 0.02 and 0.005, with a sensitivity of 64% and specificity of 57% for both parameters. 

Cats with sepsis had a significantly higher prevalence of neutrophilic toxicity than SIRS cats (85.3% vs. 14.7%, *p* = 0.0005; OR 5.6 95%CI 2.1–14.7).

In SIRS and sepsis groups, 7 (13%) and 31 cats (39.2%), respectively, died during hospitalization, but none of them was euthanized. Descriptive statistics of leukocyte ratios in survivors and non-survivors are reported in Table 2. Based on the multivariable logistic regression model, NLR resulted in the only parameter associated with mortality in SIRS and septic cats.

## 4. Discussion

The present study investigated the usefulness of leukocyte ratios to discriminate between feline SIRS and sepsis. The NLR and the other two leukocyte ratios (BLR and BNLR) as diagnostic markers demonstrated to differ between SIRS and septic cats and healthy cats. Although the prognostic significance of BLR and BNLR remains questionable, the NLR seems to be related to the mortality in SIRS and septic cats. 

We evaluated these parameters in feline SIRS and sepsis because they have demonstrated their diagnostic and prognostic utility in both humans [12,13,14,15,16,17] and dogs [3,4,5]. 

The NLR resulted able to discriminate cats with SIRS/sepsis from healthy cats. This novel finding in feline medicine may be compared only to canine studies in veterinary medicine that investigated NLR in inflammation and sepsis [3,4,5,21]. In veterinary medicine, the NLR was firstly investigated as a marker in various types of canine tumors [21,22,23,24,25], especially mast cell tumors, with contradictory results [22,24]. The NLR, more recently, was also studied in canine inflammatory bowel disease, oral melanoma, and perivascular tumors [25,26,27]. In canine SIRS and sepsis, the NLR was firstly studied in patients affected by septic peritonitis [3]. This study compared the NLR in a population of septic and non-septic SIRS compared to healthy controls. In this study, the authors found that an NLR ≥ 6 had a sensitivity and specificity of about 85% to identify dogs with systemic inflammation, although, as in our study, it cannot distinguish septic and non-septic dogs [3]. More recently, two studies by Pierini et al. [4,5] studied the canine NLR in septic and non-septic SIRS [4] and sepsis [5]. In both these reports, the NLR was investigated in dogs with SIRS and sepsis, and the results were significantly lower in the septic dogs compared to the non-septic group [4,5]. However, none of the studies found a significant association between the NLR and mortality [4,5]. Even if our data cannot be directly compared with studies based on dogs, it seems that the NLR may be a reliable marker to identify SIRS/sepsis also in cats. 

Additionally, although this result might be interested with caution based on ROC results, the BLR and BNLR resulted significantly different between healthy and sick cats and between SIRS and sepsis groups. This finding may be easily explained because healthy cats did not normally have leukocytosis, except for a mild increase of WBC linked to the stress/fear reaction [20], and, in addition, they did not show any circulating band neutrophils [20]. Indeed, band neutrophils are released into the circulation only when circulating, and the marginated neutrophil pool is exhausted, or there is a high demand on the neutrophil pool [20,28]. Due to this reason, we decided to evaluate these two ratios because they may be the feature of more severe inflammation, and they may help clinicians to better understand the inflammation pattern and decide further diagnostic investigations/monitoring.

Another finding is the higher prevalence of neutrophilic toxicity in cats with sepsis compared to the SIRS group. This result agrees with what is currently reported in feline medicine, where cats with toxic neutrophils had a significantly higher prevalence of systemic infectious conditions [29]. The presence of toxic neutrophils in peripheral blood may precede changes in the leukogram, and their presence may be used as an early marker of disease [20,29]. 

In the present study, NLR resulted significantly associated with mortality in sick cats, although its clinical relevance deserves additional studies. This association is a novel finding in cats, and this data may only be compared with human and canine studies. As aforementioned, the NLR is a well-studied diagnostic tool in human sepsis [12,13,14,15,16,17]. In all the studies, NLR resulted in a useful marker to predict sepsis, being higher in patients with sepsis. NLR has been shown as an independent predictor of death in critically ill septic patients [12,13,14,15,16,17]. On the same topic in dogs, only three studies were performed [3,4,5]. The NLR was firstly studied in dogs with and without septic peritonitis, and according to our findings, Hodgson and colleagues showed that NLR was significantly higher in dogs with SIRS, including both septic and non-septic cases than healthy dogs [3]. However, the NLR resulted not statistically associated with mortality in dogs with sepsis [3]. More recently, NLR was showed to be lower in septic dogs compared to a group of SIRS dogs [4,5], even if it was not highlighted as a predictor of death in the septic group [5]. Our data cannot be compared with canine studies, since it has been demonstrated that canine and feline leukocyte response during SIRS and, especially in sepsis, may be different [19,28,29]. The discrepancies between our findings and the canine and human studies may be related to the aforementioned differences between the proportion of circulating/marginated leukocytes pools [20,28]. In fact, the feline leukocyte poll is 2–3 times larger than human and canine ones. For this reason, leukopenia, especially neutropenia (and therefore, a lower NLR), may occur infrequently in cats than in humans and dogs [1,20,28].

This study has several limitations. Firstly, two SIRS criteria have been proposed in cats [6,7], and there are not many studies that considered them; thus, we cannot exclude that the chosen SIRS criteria are actually the most efficient. Secondly, even if we identified cats in the sepsis group based on the presence of a septic focus (evidence of bacteria in cytology and/or positive bacterial culture) with the concurrent presence of SIRS criteria, not all SIRS cats had the evidence of an absence of infection. Thus, a possible overlap of the SIRS and sepsis groups cannot be excluded. In addition, due to the retrospective nature of the present study, a clinical severity evaluation and stratification were not achievable. The use of a severity score might help to understand if there is a relationship between leukocyte ratios and disease severity. Another limitation is linked to the unknown influence of stress on NLR in cats. In dogs, NLR has been recently demonstrated to be influenced by stress in a cohort of dogs with road-transport induced stress [30]. 

## 5. Conclusions

This is the first report of the possible usefulness of NLR and other leukocyte ratios in feline patients. All the leukocyte ratios (NLR, BLR and BNLR) may be useful in the establishment of a SIRS/sepsis diagnosis. In addition, differences in BLR and BNLR deserve further studies in SIRS and septic cats. Lastly, even if other studies are warranted, the NLR may be used as a prognostic marker in feline SIRS and sepsis. 

## Figures and Tables

**Table 1:** Leukocyte ratios in healthy, systemic inflammatory response syndrome (SIRS) and sepsis groups. Data are presented as median and interquartile range (IQR) in brackets.

Parameter	Groups			*p*-Value
	Healthy (*n* = 76)	SIRS (*n* = 54)	Sepsis (*n* = 79)	
NLR	1.9 (1.4)	8.9 (13.2) ^1^	9 (18.9) ^1^	<0.0001
BLR	0 (0)	0 (1) ^1^	0.4 (2.5) ^1,2^	<0.0001
BNLR	0 (0)	0 (0.07) ^1^	0.3 (0.2) ^1,3^	<0.0001

NLR, Neutrophil-to-lymphocyte ratio; BLR, Band neutrophil-to-lymphocyte ratio; BNLR, Band neutrophils-to-segmented neutrophils-to-lymphocytes ratio. ^1^ Significantly different from healthy; *p* < 0.0001. ^2^ Significantly different from SIRS; *p* = 0.025. ^3^ Significantly different from SIRS; *p* = 0.014.

**Table 2:** Leukocyte ratios in survivors and non-survivors (*n* = 133; SIRS + sepsis groups). Data are presented as median and interquartile range (IQR) in brackets.

Parameter	Survivors (*n* = 95)	Non-Survivors (*n* = 38)	*p*-Value	Odds Ratio	95%CI Odds Ratio
Neutrophilic toxicity	20/95 (21%)	13/38 (34%)	0.06	0.46	0.17–1.04
NLR	8.6 (11.5)	10.4 (40.4)	0.02	1.02	1–1.04
BLR	0.07 (1.3)	0.46 (2.7)	0.77	1.03	0.84–1.2
BNLR	0.01 (0.08)	0.03 (0.17)	0.33	0.6	0.25–1.02

NLR, Neutrophil-to-lymphocyte ratio; BLR, Band neutrophil-to-lymphocyte ratio; BNLR, Band neutrophil-to-neutrophil-to-lymphocyte ratio.

## Data Availability

The data presented in this study are available on reasonable request from the corresponding author. The data are not publicly available due to other project involvement.
